# Fat intravasation, fat emboli and fat embolism syndrome in adult major trauma patients with intraosseous catheters: a systematic review

**DOI:** 10.1136/military-2023-002645

**Published:** 2024-05-17

**Authors:** Matt Ellington, O Hibberd, C Aylwin

**Affiliations:** 1 Department of Haematology, University of Cambridge, Cambridge, UK; 2 254 MMR, Royal Army Medical Corps, Cambridge, UK; 3 Blizard Institute, Centre for Trauma Sciences, Queen Mary University of London, London, UK; 4 Emergency and Urgent Care Research in Cambridge (EURECA), PACE Section, Department of Medicine, University of Cambridge, Cambridge, UK; 5 Centre for Trauma Sciences, Blizard Institute, QMUL, London, UK

**Keywords:** ACCIDENT & EMERGENCY MEDICINE, Adult intensive & critical care, Trauma management, TRAUMA MANAGEMENT

## Abstract

**ABSTRACT:**

**Introduction:**

Intraosseous (IO) administration of medication, fluids and blood products is accepted practice for critically injured patients in whom intravenous access is not immediately available. However, there are concerns that high intramedullary pressures resulting from IO infusion may cause bone marrow intravasation and subsequent fat embolisation. The aim of this systematic review is to synthesise the existing evidence describing fat intravasation, fat embolism and fat embolism syndrome (FES) following IO infusion.

**Methods:**

A systematic search of CINAHL, MEDLINE and Embase was undertaken using the search terms “intraosseous”, “fat embolism”, “fat intravasation” and “fat embolism syndrome”. Two authors independently screened abstracts and full texts, against eligibility criteria and assessed risk of bias. A grey literature search (including references) was undertaken. Inclusion criteria were: all human and animal studies reporting novel data on IO-associated fat emboli. This systematic review was conducted in accordance with the Preferred Reporting Items for Systematic Reviews and Meta-Analysis.

**Results:**

22 papers were identified from the search, with a further 5 found from reference lists. N=7 full papers met inclusion criteria. These papers were all translational animal studies. The overall risk of bias was high. Studies demonstrated that fat intravasation and fat embolisation are near universal after IO infusion, but of uncertain clinical significance. The initial IO flush appears to cause the highest intramedullary pressure and highest chance of fat intravasation and embolisation. No conclusions could be drawn on FES.

**Conclusions:**

IO catheters remain a useful intervention in the armamentarium of trauma clinicians. Although their use is widely accepted, there is a paucity of evidence investigating fat embolisation in IO infusions. Despite this, pulmonary fat emboli after IO infusion are very common. The existing data are of low quality with a high risk of bias. More research is needed to address this important subject.

**PROSPERO registration number:**

CRD42023399333.

**WHAT IS ALREADY KNOWN ON THIS TOPIC**

**WHAT THIS STUDY ADDS**

- This systematic review presents a narrative synthesis of the available evidence, identifying factors that may lead to fat intravasation, embolisation and fat embolisation syndrome.

**HOW THIS STUDY MIGHT AFFECT RESEARCH, PRACTICE OR POLICY**

- This review concludes that fat intravasation is near universal with intraosseous catheter usage, and that the initial flush appears to be the aspect of intraosseous catheter insertion associated with the highest likelihood of fat embolism.

## Introduction

 The leading cause of potentially survivable traumatic death, in both civilian and military settings, is haemorrhage.^1^ This accounts for 30–40% of total trauma deaths.^2^ Expedient vascular access is of critical importance in hypovolaemic trauma patients.^3^ When peripheral venous access is not immediately available, intraosseous (IO) catheters can be used as a rapidly achievable, non-collapsible intravascular route for initiating resuscitation and administering life-saving medications.^4^ IO access is twice as likely to be successful as peripheral cannulation,^3^ and is faster than both central and peripheral intravenous access.^5^ Accordingly, IO access is widely advocated in critically injured patients as a rescue technique when peripheral intravenous access is difficult or unsuccessful.^6^

More than a million IO cannulas have been used in the last decade.^7^ IO access is advocated by numerous life support courses, in both civilian and military sectors^8^ (ALS, ATLS, APLS, Battlefield Advanced Trauma Life Support and Tactical Combat Critical Care).^9^,^13^ IO access is described as a safe and reliable intervention^14^; however, the evidence base and utility of IO access remain disputed.^15^

IO success rates are reportedly between 80% and 95% in most series,^8 16 17^ but there is a paucity of data regarding complications and sequelae of IO access. Major complications are rare, but include device failure, malpositioning, osteomyelitis, compartment syndrome and bone fracture.^16 17^ Importantly, there is clinical concern regarding the risk of bone marrow entering the pulmonary (and systemic) circulation during IO infusion (fat intravasation). It is suggested that changes in intramedullary pressure during IO procedures may be implicated in the intravasation of bone marrow.^18^ The displaced bone marrow can then travel in the venous system (fat embolus) to the pulmonary or systemic vasculature, where it becomes lodged. IO infusions have been shown in animal studies to cause both pulmonary and systemic fat emboli,^19^ and fat emboli are a potentially devastating phenomenon in critically injured patients.^20^ As early as 1989, Orlowski *et al* reported fat embolism being a universal finding (at postmortem) following IO infusions in a translational animal model.^21^ Fat emboli can manifest significant clinical sequelae, known as fat embolism syndrome (FES). FES usually has a gradual onset (12–36 hours post-injury) and consists of hypoxia, neurological symptoms, fever and a petechial rash.^22^ The lung dysfunction in FES is clinically indistinguishable from acute respiratory distress syndrome.^23^

There are several proposed theories as to the pathophysiological mechanism of FES. One of the most widely accepted, the biochemical inflammatory theory, proposes that the displaced bone marrow induces a systemic inflammatory response mediated by elevated levels of free fatty acids and inflammatory mediators. Free fatty acids have also been shown to induce inflammation within the lungs.^23^ FES causes an exaggerated inflammatory response.^24^

There has been recent interest in systemic inflammation and multiple organ dysfunction syndrome (MODS) following major trauma. In a recent prospective observational study conducted in the UK, major trauma patients without MODS had significantly lower mortality, ventilator days, intensive care unit days and total hospital stay duration, compared with those with MODS. The mortality rate for patients with MODS was 44 times higher than those without MODS. There was unfortunately no subgroup analysis for patients who had had IO access performed.^25^

These concerns are however derived from a small evidence base, predominantly conducted on translational animal models^21^ or from postmortem reports from paediatric patients,^26^ with limited translatability to adult trauma patients. This systematic review has been conducted to better understand the likelihood of fat intravasation, embolisation and FES, in adult trauma patients with IO access. The primary aim of this review is to characterise the likelihood of fat intravasation, pulmonary and systemic fat emboli, and FES, after IO access, in major trauma patients.

## Methods

A systematic search was conducted in accordance with the Preferred Reporting Items for Systematic Reviews and Meta-Analyses guidelines.^27^ Search terms were “intraosseous”, “fat intravasation”, “fat embolism” and “fat embolism syndrome”. The full search strategy is presented in online supplemental appendix 1. Relevant abstracts identified in the search were reviewed against the inclusion criteria independently by two authors. Full-text articles were retrieved, which were then independently reviewed by two authors. The protocol for this systematic review was prospectively registered with the University of York Centre for Reviews and Dissemination and the National Institute for Health Research PROSPERO database (registration number: CRD42023399333).^28^

Three databases were used for the search (Medline on EBSCO, CINAHL on EBSCO, EMBASE on Ovid) which was conducted from inception to January 2023. Included study designs were human studies and translational animal studies, limited to those reported in English language only. The inclusion and exclusion criteria are summarised in table 1.

**Table 1:** Inclusion and exclusion criteria

	Inclusion	Exclusion
Population	Major trauma (ISS >15) patients	Trauma patients with ISS <15
Intervention/exposure	IO catheter placement	
Comparator	IV catheter placement	
Outcome	Fat intravasation, emboli (histologically confirmed or otherwise clearly defined in article) or FES (clinically suspected or otherwise defined)	
Study design/methodology	Clinical trials (randomised and non-randomised), observational studies (cohort and case–controlled), case series and literature reviews. Military, civilian and translational animal studies all included.	Conference abstracts, case reports, case series, systematic reviews/meta-analyses, opinion articles and case reports
Other criteria		Articles not published in English language

FES, fat embolism syndrome; IO, intraosseous; ISS, Injury Severity Score; IV, intravenous.

To be confident of having complete capture of the literature, the reference lists of identified articles were reviewed. A grey literature search was conducted to identify any novel data. Where necessary, corresponding authors were contacted for any further additional information.

Data extraction was performed independently by two authors, after reviewing the selected full-text articles. Any discrepancies were to be adjudicated by a third author, although this was not necessary. A bespoke data sheet (Microsoft Corporation, Microsoft Excel, internet (2016)) was used to record pertinent study information and assess the risk of bias of included studies. Extracted data included: study design, population studied, study methodology, infusion and flush method, bone used, outcome measure and results. The primary outcomes for this systematic review were fat intravasation, fat emboli and FES.

Quantifying statistical heterogeneity of included studies was attempted using Review Manager software (RevMan; V.5.3). Assessment of heterogeneity was planned to be completed using the I^2^ statistic. The study protocol stated a threshold for statistical heterogeneity of 40%.

### Assessment of methodological quality and study risk of bias assessment

Risk of bias screening for each included study was undertaken using the Risk of Bias in Non-Randomised Studies–Interventions (ROBINS-I) tool.^29^ This is the preferred tool for risk of bias screening in the Cochrane Handbook for Systematic Reviews of Interventions.^30^ Bias scoring was undertaken by two authors working independently. Any disagreements were to be adjudicated by a third author, although this was not necessary. The ROBINS-I tool assesses the following domains: confounding, selection bias, bias in measurement classification of interventions, bias due to deviations from intended interventions, bias due to missing data, bias in measurement of outcomes and bias in selection of the reported result.

All studies included in this review were assessed in accordance with the Oxford Centre of Evidence-Based Medicine framework.^31^ Each study was assigned a level of evidence score on a 5-point scale.

## Results

**Figure 1:**
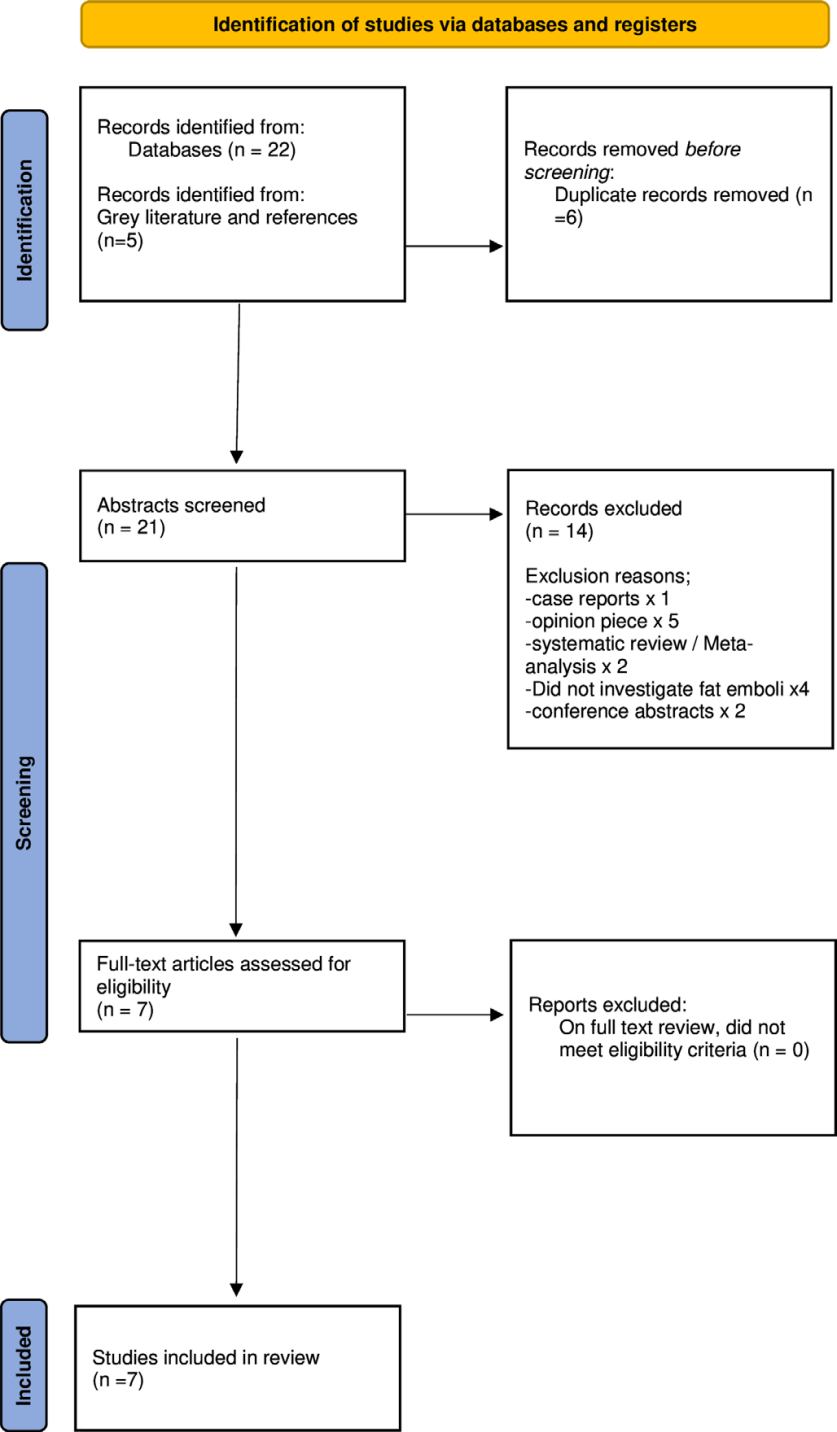
Preferred Reporting Items for Systematic Reviews and Meta-Analysis flow diagram.

The search strategy identified n=22 studies. A further n=5 studies were identified from searches of grey literature and reference lists. Six duplicate results were removed, leaving n=21 unique studies. Following abstract screening, 14 papers were excluded that did not meet inclusion criteria (a case report, five opinion pieces, two systematic reviews, four papers not directly investigating fat emboli and two conference abstracts). The remaining n=7 papers subsequently underwent full-text review, with no further exclusions. This systematic review therefore included n=7 studies. The flow diagram is presented in figure 1.

These papers were all large translational animal studies performed on swine. The pertinent study findings are summarised in table 2.

**Table 2:** Summary of studies included in this systematic review

Authors	OCEBM level of evidence	Study population	Bone used	Flush used	Infusion method	Observation time post-infusion	Outcome measured and definition	Results
Fiallos *et al*^26^	3	33 mixed-breed piglets	Tibia	3 mL NaCl	Pressure bag @300 mm Hg	0 min	Buffy coat examination and postmortem lung histology	Pulmonary fat embolism in all lung samples
Kristiansen *et al*^19^	3	28 piglets (3–4 weeks old)	Tibia	10 mL NaCl	Pressure bag @ 300 mm Hg	5 hours	Continuous Transthoracic Echocardiography and postmortem lung histology	Pulmonary fat embolism in all lung samples. 7/21 also had coronary fat emboli.
Rubal and Andrews^34^	3	35 swine, mean age 4 months	Tibia	Various	Various	24 hours	Vascular ultrasound imaging with lipophilic fluoroprobe	Fat intravasation was observed during all IO infusions. Initial flush a significant factor.
Plewa *et al*^32^	3	16 swine (mean age 2 months)	Tibia	1 mL NaCl	3-way tap and ‘maximal manual pressure’	48 hours	Postmortem lung histology	No fat emboli
Sulava *et al*^3^	3	48 adult swine	Various	3 mL NaCl	Pressure bag @360 mm Hg	1 hour	Postmortem lung histology	80% had fat emboli, 15% had pulmonary oedema
Hasan *et al*^33^	3	28 mixed-breed piglets	Tibia	3 mL NaCl	Various including pressure bag @300 mm Hg	0 hour	Buffy coat examination and postmortem lung histology	Method of IO fluid administration does not influence the number of emboli
Auten *et al*^4^	3	36 adult female swine	Humerus	10 mL NaCl	Various including pressure bag (300 and 360 mm Hg) and rapid infuser (alarm limit 300 mm Hg)	1 hour	Postmortem lung histology	All but 1 pulmonary specimens had fat embolism

IO, intraosseous; NaCl, sodium chloride; OCEBM, Oxford Centre of Evidence-Based Medicine.

One study investigated FES (rather than fat intravasation or embolism), but found that on histopathological inspection, all 16 lung specimens were free from fat emboli or inflammation.^32^

The other six papers reported positive findings. Pulmonary fat emboli were very common, being found in 30%,^33^ 75%,^3^ 97%^4^ and 100%^19^ of lung samples examined. Two studies looked for emboli which occlude pulmonary vessels, and neither found any evidence of occlusion.^3 4^ Kristiansen *et al*^19^ also performed postmortem cardiac histology and found that 33% of 21 swine have fat emboli in their coronary arteries. Plewa *et al*^32^ were the only study to specifically investigate FES. They used immature swine that underwent autologous whole blood transfusion via IO and intravenous cannulae, with the animals euthanised and lung samples collected 48 hours afterwards. They found no evidence of either emboli or FES. However, the mean age of the swine was only 2 months old, the flush process was not described and the mean infusion flow rate was clinically insufficient for trauma resuscitation (only 21 mL/min (IO) and 35 mL/min (intravenously), all of which limit the translatability and applicability of these results to adult trauma patients.

There was significant variation in study methodologies; five studies used piglets,^1926 32^,^34^ with only two studies using skeletally mature swine.^3 4^ The composition of IO infusion also varied significantly; three studies used autologous warm fresh whole blood, with the others using either lactated ringer’s solution, saline or commonly used emergency drugs. The only study to directly compare IO versus intravenous infusions used immature swine.^32^ Fat embolisation was inconsistently defined and measured across the seven studies. The majority of methodologies examined lung parenchymal samples that were taken for postmortem histological analysis to quantify fat emboli microscopically. Other methods included buffy coat examination and continuous ultrasonographic imaging. The timing of post-infusion observation and lung sample collection varied widely, with some animals euthanised for lung biopsy immediately, some at 60 min, 300 min, 24 hours and 48 hours post-IO infusion or transfusion.

Although some studies attempted to model hypovolaemia (by controlled exsanguination), no studies attempted to model both hypovolaemia and concurrent tissue injury as seen in major trauma. This tissue injury and resulting immune response could contribute towards inflammatory sequelae of fat emboli and FES/MODS.

The study protocol stated a threshold for statistical heterogeneity using the I^2^ test of 40%. During the data analysis, however, it became apparent that none of the included studies shared the same intervention and comparator; therefore, it was not possible to quantify heterogeneity, and a narrative synthesis of the reported study findings was instead performed.

The detailed risk of bias assessments for individual studies are presented in table 3. Overall, the risk of bias was judged to be high, and no study had a risk of bias deemed less than moderate. Fiallos *et al* ^26^ investigated IO infusions with concurrent cardiopulmonary resuscitation (CPR). This was judged a critical confounder as it would have been impossible to distinguish fat emboli associated with IO catheters, from fat emboli from fractured ribs during CPR. Kristiansen *et al*^19^ excluded one animal during the study period which suffered a cardiac arrest. It was not clear if this cardiac arrest was temporally associated with the IO procedure (which could have been due to fat emboli), and no confirmation of cause of arrest or pulmonary histology was performed. Due to deviation from the intended intervention and bias from missing data, this study was deemed at serious risk of bias. For four studies,^3 19 32 34^ lack of blinding of outcome assessors meant that they could have known the intervention received, and therefore were classed as moderate risk of bias.

**Table 3:** The risk of bias assessment using the ROBINS-I tool for included studies

	Domain one	Domain two	Domain three	Domain four	Domain five	Domain six	Domain seven
Fiallos *et al*^26^	Critical	No information	Low	Low	Low	Low	Low
Kristiansen *et al*^19^	Low	Low	Low	Serious	Serious	Moderate	Low
Rubal and Andrews^34^	Low	Low	Low	Low	Low	Moderate	Low
Plewa *et al*^32^	Low	Low	Low	Low	Low	Moderate	Low
Sulava *et al*^3^	Low	Low	Low	Low	Low	Moderate	Low
Hasan *et al*^33^	Low	Low	Low	Low	Low	Low	Moderate
Auten *et al*^4^	Low	Low	Low	Low	Low	Low	Moderate

ROBINS-I, Risk of Bias in Non-Randomised Studies–Interventions.

## Discussion

Pulmonary sequelae of IO infusion are understudied, poorly understood and continue to be a safety concern associated with IO usage. This systematic review of seven studies investigating fat intravasation, embolisation and FES revealed wide heterogeneity in study design, methodology, study population, intervention and outcome measurement. Overall, fat intravasation and fat embolism in the included studies were extremely common. Despite the methodological heterogeneity, of a total of 224 swine across the seven studies, 186 (83%) had proven pulmonary fat emboli.

Despite this methodological heterogeneity and a paucity of high-quality evidence, this review has identified two key factors affecting fat intravasation, fat embolisation and FES after IO infusion: the bone used, and the flush method used, for infusion.

### Bone

Of the seven animal studies included in this review, only two were conducted on skeletally mature swine. The remaining five studies were conducted on swine less than 4 months old. There are changes in bone density throughout life and differences between male and female patients. The bone density of a healthy military-aged human (20–40 years old) is approximately double that of the paediatric population.^15^ Other recent reviews have also noted the issues of conducting IO research on immature swine in terms of their translatability to adult military trauma patient population.^35^

There are two key reasons why immature swine bone may not be representative of typical adult trauma patients. First, Darcy’s law describes the two aspects governing IO flow rate as bone density and fluid viscosity.^36^ Resultantly, lower pressure is needed to infuse fluids into immature bone, and higher infusion pressures are needed to maintain infusion rates as bone density increases with age.^37^ Additionally, the composition of the intramedullary space also changes with age. Due to the incomplete transformation of red marrow into adipose cells, skeletally immature humans^33^ and animals^34^ may have less fat in their bone marrow and may be less likely to shed emboli.

The only two studies using mature swine^3 4^ (with bone density comparable with adult major trauma patients) both found widespread pulmonary fat emboli on histological examination of the lungs.

It is also prudent to note that data are lacking for female trauma patients. There were no studies designed to investigate fat intravasation, embolisation or FES in female trauma patients. Females are known to have differences in average bone densities compared with males^38^ and although a relative minority, female trauma patients are a significant and understudied group in current literature.

### Flush technique

Of the seven studies included in this review, one used a 1 mL flush, three used a 3 mL flush, two used a 10 mL flush and one study used various volumes. None of the studies described their flush technique in detail. Hasan *et al*^33^ noted the number of emboli does not correspond to infusion pressure, infusion rate or total volume of fluid, but they did not consider the effect of the initial flush on fat embolisation rates. It may indeed be the initial flush that generates the biggest changes in intramedullary pressure and subsequent embolisation of fat. Only recently has the contribution of the initial IO flush been implicated in fat intravasation and embolisation.^39^ Given the methodological heterogeneity of these seven studies, it is surprising that the results are so consistent. Fat emboli originating from the initial flush, rather than infusion, could explain this.

In one study, one animal had acute hypoxaemia, hypocapnia and cardiovascular instability with rapid flush (pressure >2000 mm Hg).^34^ The authors concluded that the initial flush contributed more bone marrow intravasation than IO infusions (at constant pressures <300 mm Hg). Rubal and Andrews^34^ used continuous vascular ultrasound imaging with a lipophilic fluoroprobe to assess for fat emboli in their study. They observed an 11-fold increase in fat fluorescence during the flush.

A median IO pressure of 903 mm Hg during the flush of an IO catheter has been demonstrated.^18^ This pressure is significantly higher than pressures seen during infusion with rapid infusers or pressure bags (around 300 mm Hg) and interventional orthopaedic procedures (around 400 mm Hg),^40^ which are traditionally considered high risk for fat intravasation and embolisation. Furthermore, studies investigating IO flush technique have found high variability in emergency department clinicians’ clinical practice on flushes, 15% of which exceed 2000 mm Hg.^18^

### Fat embolism syndrome

FES is a clinical diagnosis based on gradual onset of fever, hypoxia, neurological symptoms and a petechial rash, usually occurring 12–36 hours after the fat embolus.^22^ Given the logistical difficulties of measuring a clinical diagnosis and neurological symptoms in anaesthetised swine, it is unsurprising that very few study methodologies did so. Of the seven included studies, two euthanised the swine at the end of the IO infusion,^26 33^ a further two studies euthanised the animals after a 60 min observation period^3 4^ and one euthanised at 5 hours.^19^ Of the two studies to monitor the animals long enough post-infusion for FES to manifest, one did not investigate FES,^34^ and the other was the only study not to find any pulmonary fat emboli at all.^32^

This lack of adequate follow-up time post-intervention was noted by Hasan *et al*^33^ who stated ‘because FES usually occurs 48–72 hours after injury, to determine the clinical significance of fat embolisation with IO infusions, it will be necessary to assess clinical and laboratory parameters of the animals over a longer time’. Overall, from these data and the lack of appropriate follow-up, it is not possible to comment on FES following IO infusion.

IO access remains an important aspect to the clinical management of adults with major trauma; its utility for administration of fluids and medications is proven, established and accepted practice in major trauma. The safety and sequelae of IO infusion however are understudied, poorly described and poorly understood.

### Limitations

There are several limitations to this systematic review.

The main limitations of this review are the low number of studies and their high risk of bias. Despite good applicability of translational animal models, there remain anatomical and physiological differences between swine and humans that limit the applicability of study findings to the human trauma population. Eligible studies were limited in both numbers and quality. Data analysis was difficult due to methodological heterogeneity which meant that a meta-analysis could not be performed.

Furthermore, these studies were conducted using a normovolaemic or hypovolaemic trauma model. These animals did not have any concurrent tissue injuries (such as seen in significant trauma) which may have a significant impact on immunogenic and inflammatory processes. The vasodilating effect of inhalational anaesthetic agents used to maintain anaesthesia in the study subjects may also have affected the required driving pressure for IO infusion and likelihood of embolisation.

### Areas for future research

This systematic review has identified the need for high-quality research on this crucial research question. Future research should use methodologies which allow longitudinal observation of inflammatory sequelae of fat emboli in humans, while controlling for obvious confounders for marrow intravasation such as fractured bones.

## Conclusions

IO catheters remain a useful intervention in the armamentarium of trauma clinicians. Although their use is widely accepted, there is a paucity of evidence exploring the likelihood of fat embolisation in IO infusions. Despite this, pulmonary fat emboli after IO infusion are common. The existing data are of low quality with a high risk of bias. More research is needed to quantify the immunological sequelae of fat embolism/FES after IO infusion in adults with major traumatic injuries.

## Supplementary material

10.1136/military-2023-002645 online supplemental appendix 1

## Data Availability

Data are available upon reasonable request. All data relevant to the study are included in the article or uploaded as supplemental information.
