# Supplementary material for: Fat intravasation, fat emboli and fat embolism syndrome in adult major trauma patients with intraosseous catheters: a systematic review
Source: BMJ Mil Health. 2024 May 17;172(1):e002645. doi: 10.1136/military-2023-002645 (PMC12911625; doi:10.1136/military-2023-002645)
Supplement: online supplemental appendix 1 [file military-172-1-s001.pdf]

Appendix 1 – search strategy

Search strategies

Number of articles retrieved

Database	Total retrieved	Total included
Medline	10	10
CINAHL	14	10
EMBASE	4	2

Total included from all databases	28
Total duplicates	6
Total included in the results	22

Limiters

English language

CINAHL

**Search ID#**	**Search Terms**	**Last Run Via**	**Results**
S32	S18 AND S31	Interface - EBSCOhost Research Databases Search Screen - Advanced Search Database - CINAHL	5
S31	S29 OR S30	Interface - EBSCOhost Research Databases Search Screen - Advanced Search Database - CINAHL	9,052
S30	AB cannula*	Interface - EBSCOhost	7,473

		Research Databases Search Screen - Advanced Search Database - CINAHL	
S29	TI cannula*	Interface - EBSCOhost Research Databases Search Screen - Advanced Search Database - CINAHL	3,387
S28	S18 AND S27	Interface - EBSCOhost Research Databases Search Screen - Advanced Search Database - CINAHL	8
S27	S25 OR S26	Interface - EBSCOhost Research Databases Search Screen - Advanced Search Database - CINAHL	54,212
S26	AB catheter*	Interface - EBSCOhost Research Databases Search Screen - Advanced Search Database - CINAHL	42,712
S25	TI catheter*	Interface - EBSCOhost Research Databases Search Screen - Advanced Search Database - CINAHL	23,964
S24	S6 AND S23	Interface - EBSCOhost	3

		Research Databases Search Screen - Advanced Search Database - CINAHL	
S23	S12 OR S15	Interface - EBSCOhost Research Databases Search Screen - Advanced Search Database - CINAHL	1,394
S22	S15 AND S18	Interface - EBSCOhost Research Databases Search Screen - Advanced Search Database - CINAHL	1
S21	S12 AND S18	Interface - EBSCOhost Research Databases Search Screen - Advanced Search Database - CINAHL	0
S20	S9 AND S12 AND S18	Interface - EBSCOhost Research Databases Search Screen - Advanced Search Database - CINAHL	0
S19	S3 AND S6 AND S12 AND S18	Interface - EBSCOhost Research Databases Search Screen - Advanced Search Database - CINAHL	0
S18	S16 OR S17	Interface - EBSCOhost	285

		Research Databases Search Screen - Advanced Search Database - CINAHL	
S17	AB fat intravasation or fat emboli or fat embolism syndrome	Interface - EBSCOhost Research Databases Search Screen - Advanced Search Database - CINAHL	197
S16	TI fat intravasation or fat emboli or fat embolism syndrome	Interface - EBSCOhost Research Databases Search Screen - Advanced Search Database - CINAHL	174
S15	S13 OR S14	Interface - EBSCOhost Research Databases Search Screen - Advanced Search Database - CINAHL	1,358
S14	AB intravenous catheter	Interface - EBSCOhost Research Databases Search Screen - Advanced Search Database - CINAHL	1,087
S13	TI intravenous catheter	Interface - EBSCOhost Research Databases Search Screen - Advanced Search Database - CINAHL	543
S12	S10 OR S11	Interface - EBSCOhost	43

		Research Databases Search Screen - Advanced Search Database - CINAHL	
S11	AB intraosseous catheter*	Interface - EBSCOhost Research Databases Search Screen - Advanced Search Database - CINAHL	32
S10	TI intraosseous catheter*	Interface - EBSCOhost Research Databases Search Screen - Advanced Search Database - CINAHL	16
S9	S7 OR S8	Interface - EBSCOhost Research Databases Search Screen - Advanced Search Database - CINAHL	167,698
S8	AB increased risk or higher risk	Interface - EBSCOhost Research Databases Search Screen - Advanced Search Database - CINAHL	161,690
S7	TI increased risk or higher risk	Interface - EBSCOhost Research Databases Search Screen - Advanced Search Database - CINAHL	9,313
S6	S4 OR S5	Interface - EBSCOhost	3,176

		Research Databases Search Screen - Advanced Search Database - CINAHL	
S5	AB major trauma	Interface - EBSCOhost Research Databases Search Screen - Advanced Search Database - CINAHL	2,703
S4	TI major trauma	Interface - EBSCOhost Research Databases Search Screen - Advanced Search Database - CINAHL	956
S3	S1 OR S2	Interface - EBSCOhost Research Databases Search Screen - Advanced Search Database - CINAHL	433,219
S2	AB adult	Interface - EBSCOhost Research Databases Search Screen - Advanced Search Database - CINAHL	360,488
S1	TI adult	Interface - EBSCOhost Research Databases Search Screen - Advanced Search Database - CINAHL	173,043

MEDLINE

Search ID#	Search Terms	Last Run Via	Results
S28	S18 AND S27	Interface - EBSCOhost Research Databases Search Screen - Advanced Search Database - MEDLINE	12
S27	S25 OR S26	Interface - EBSCOhost Research Databases Search Screen - Advanced Search Database - MEDLINE	21,377
S26	AB cannula	Interface - EBSCOhost Research Databases Search Screen - Advanced Search Database - MEDLINE	19,661
S25	TI cannula	Interface - EBSCOhost Research Databases Search Screen - Advanced Search Database - MEDLINE	4,077
S24	S6 AND S23	Interface - EBSCOhost Research Databases Search Screen - Advanced Search	1

		Database - MEDLINE	
S23	S12 AND S15	Interface - EBSCOhost Research Databases Search Screen - Advanced Search Database - MEDLINE	174
S22	S15 AND S18	Interface - EBSCOhost Research Databases Search Screen - Advanced Search Database - MEDLINE	4
S21	S12 AND S18	Interface - EBSCOhost Research Databases Search Screen - Advanced Search Database - MEDLINE	3
S20	S9 AND S12 AND S18	Interface - EBSCOhost Research Databases Search Screen - Advanced Search Database - MEDLINE	0
S19	S3 AND S6 AND S12 AND S18	Interface - EBSCOhost Research Databases Search Screen - Advanced Search Database - MEDLINE	0

S18	S16 OR S17	Interface - EBSCOhost Research Databases Search Screen - Advanced Search Database - MEDLINE	1,174
S17	AB fat intravasation or fat emboli or fat embolism syndrome	Interface - EBSCOhost Research Databases Search Screen - Advanced Search Database - MEDLINE	881
S16	TI fat intravasation or fat emboli or fat embolism syndrome	Interface - EBSCOhost Research Databases Search Screen - Advanced Search Database - MEDLINE	642
S15	S13 OR S14	Interface - EBSCOhost Research Databases Search Screen - Advanced Search Database - MEDLINE	5,530
S14	AB intravenous catheter or intravenous access	Interface - EBSCOhost Research Databases Search Screen - Advanced Search Database - MEDLINE	4,881
S13	TI intravenous catheter or intravenous access	Interface - EBSCOhost Research	1,338

		Databases Search Screen - Advanced Search Database - MEDLINE	
S12	S10 OR S11	Interface - EBSCOhost Research Databases Search Screen - Advanced Search Database - MEDLINE	518
S11	AB intraosseous catheter or intraosseous access	Interface - EBSCOhost Research Databases Search Screen - Advanced Search Database - MEDLINE	411
S10	TI intraosseous catheter or intraosseous access	Interface - EBSCOhost Research Databases Search Screen - Advanced Search Database - MEDLINE	241
S9	S7 OR S8	Interface - EBSCOhost Research Databases Search Screen - Advanced Search Database - MEDLINE	381,418
S8	AB increased risk	Interface - EBSCOhost Research Databases Search Screen - Advanced Search	373,425

		Database - MEDLINE	
S7	TI increased risk	Interface - EBSCOhost Research Databases Search Screen - Advanced Search Database - MEDLINE	14,937
S6	S4 OR S5	Interface - EBSCOhost Research Databases Search Screen - Advanced Search Database - MEDLINE	8,339
S5	AB major trauma	Interface - EBSCOhost Research Databases Search Screen - Advanced Search Database - MEDLINE	7,514
S4	TI major trauma	Interface - EBSCOhost Research Databases Search Screen - Advanced Search Database - MEDLINE	1,825
S3	S1 OR S2	Interface - EBSCOhost Research Databases Search Screen - Advanced Search Database - MEDLINE	415,429

S2	TI adult	Interface - EBSCOhost Research Databases Search Screen - Advanced Search Database - MEDLINE	415,429
S1	TI adult	Interface - EBSCOhost Research Databases Search Screen - Advanced Search Database - MEDLINE	415,429

EMBASE

#	Query	Results from 24 Jan 2023
1	adult.m_titl.	242,419
2	adult/	8,959,290
3	limit 2 to abstracts	8,013,946
4	1 or 3	8,115,043
5	major trauma.m_titl.	1,968
6	major trauma.mp.	6,459
7	limit 6 to abstracts	6,173
8	5 or 7	6,452
9	increased risk.m_titl.	17,590
10	increased risk.mp.	434,284
11	limit 10 to abstracts	431,507

12	intraosseous catheter.m_titl.	12
13	intraosseous catheter.mp.	46
14	limit 13 to abstracts	38
15	12 or 14	40
16	intravenous catheter.m_titl.	576
17	intravenous catheter.mp.	10,110
18	limit 17 to abstracts	8,705
19	16 or 18	8,825
20	(fat intravasation or fat emboli or fat embolism syndrome).m_titl.	692
21	(fat intravasation or fat emboli or fat embolism syndrome).mp. [mp=title, abstract, heading word, drug trade name, original title, device manufacturer, drug manufacturer, device trade name, keyword heading word, floating subheading word, candidate term word]	1,342
22	limit 21 to abstracts	1,141
23	20 or 22	1,333
24	4 and 8 and 15 and 23	0
25	9 or 11	434,282
26	4 and 8 and 15 and 23	0
27	9 or 11	434,282
28	15 and 23 and 27	0
29	15 and 23	1
30	15 and 19	5
31	cannula.m_titl.	4,611
32	cannula.mp.	42,541
33	limit 32 to abstracts	38,325
34	31 or 33	39,457
35	23 and 34	18

36	15 and 19 and 23	0
